# Puerperal Sepsis; Its Prophylaxis and Treatment

**Published:** 1894-03

**Authors:** 


**Puerperal Sepsis; its Prophylaxis and Treatment.**—Dr. Robert A. Murray read a paper with this title. Antisepsis during labor, he said, was believed to be as necessary as in surgery. Three antiseptics could be managed easily—corrosive sublimate, carbolic acid and creolin. The prophylaxis should begin with a bath and a change of clothing. The vulva especially should be cleansed carefully. A new rubber cloth should be spread over a clean sheet, and over the rubber there should be a draw-sheet. The importance of an enema should not be forgotten. A vaginal injection of a 1-to-3000 solution of corrosive sublimate, or of a two-per-cent. solution of creolin should be given before labor, but usually not after it. Creolin was to be preferred to corrosive sublimate, because it was something of a lubricant, while corrosive sublimate was distinctly astringent, and so facilitated laceration. An antiseptic pad and napkin should then be applied, and never removed except for examination, which should not be more frequent than was actually necessary. The physician's hands should be scrubbed thoroughly and immersed in a 1-to-2000 solution of creolin. The antiseptic pad should be applied again after the birth of the child and before the expulsion of the placenta. Retained membranes and pieces of placenta should be removed at once by the aseptic hand, and then an intra-uterine douche should be given. Fluid extract of ergot, in half-drachm or drachm doses, was to be recommended in the majority of cases. After urination an antiseptic solution should be applied. When there was a bad odor to the discharge a vaginal douche was to be ordered, but if the examining finger showed that the foul dis-

*This is the American Medical Association's bill, now before Congress.

charge came from the uterus, it must be thoroughly curetted and a single intra-uterine douche given. In prolonged cases instruments were to be employed. The nurse should never be allowed to examine a patient. In case of sepsis, the same general plan of antisepsis should be followed, but the vagina ought to be inspected so that diphtheritic membrane might not be carried into the uterus.—*New York Medical Journal.*

